# Peripheral decarboxylase inhibitors paradoxically induce aromatic L-amino acid decarboxylase

**DOI:** 10.1038/s41531-021-00172-z

**Published:** 2021-03-19

**Authors:** Anouke van Rumund, Lukas Pavelka, Rianne A. J. Esselink, Ben P. M. Geurtz, Ron A. Wevers, Brit Mollenhauer, Rejko Krüger, Bastiaan R. Bloem, Marcel M. Verbeek

**Affiliations:** 1 Department of Neurology, Radboud University Medical Center, Donders Institute for Brain, Cognition and Behaviour, Nijmegen, The Netherlands; 2 Radboudumc Center of Expertise for Parkinson & Movement Disorders, Nijmegen, The Netherlands; 3 Clinical and Experimental Neuroscience, Luxembourg Centre for Systems Biomedicine, University of Luxembourg, Esch-sur-Alzette, Luxembourg; 4 Parkinson’s Research Clinic, Centre Hospitalier de Luxembourg, Luxembourg, Luxembourg; 5 Department of Laboratory Medicine, Radboud University Medical Center, Nijmegen, The Netherlands; 6 Center of Parkinsonism and Movement Disorders, Paracelsus-Elena-Klinik, Kassel, Germany; 7 University Medical Center, Department of Neurology, Göttingen, Germany; 8 Transversal Translational Medicine, Luxembourg Institute of Health, Strassen, Luxembourg

**Keywords:** Parkinson's disease, Enzymes

## Abstract

Peripheral decarboxylase inhibitors (PDIs) prevent conversion of levodopa to dopamine in the blood by the enzyme aromatic L-amino acid decarboxylase (AADC). Alterations in enzyme activity may contribute to the required higher dosages of levodopa observed in many patients with Parkinson’s disease. We evaluated the effect of levodopa/PDI use on serum AADC enzyme activity. Serum AADC enzyme activity was evaluated in three independent cohorts of patients with Parkinson’s disease or parkinsonism (*n* = 301) and compared between patients on levodopa/PDI vs. patients not on this medication. AADC enzyme activity was elevated in 62% of patients on levodopa/PDI treatment, compared to 19% of patients not on levodopa/PDI (median 90 mU/L vs. 50 mU/L, *p* < 0.001). Patients with elevated AADC activity had longer disease duration and higher doses of levodopa/PDI. These findings may implicate that peripheral AADC induction could underlie a waning effect of levodopa, necessitating dose increases to maintain a sustained therapeutic effect.

## Introduction

Levodopa is the mainstay of pharmacotherapy for patients with Parkinson’s disease (PD). Levodopa is typically administered with a peripheral decarboxylase inhibitor (PDI), benserazide or carbidopa. PDIs bind irreversibly to pyridoxal-5-phospate (PLP), the active form of vitamin B6. PLP is required for the functioning of numerous enzymes and proteins. One of its many functions is to act as a coenzyme for activation of L-amino acid decarboxylase (AADC), which catalyzes the conversion of levodopa in dopamine. PDIs thus prevent peripheral conversion of levodopa to dopamine^1^, thereby diminishing side effects such as nausea or orthostatic hypotension that are caused by high systemic dopamine levels. Moreover, PDIs prolong the half-life of levodopa in blood, thereby increasing the amount of levodopa that crosses the blood–brain barrier and boosting its therapeutic efficacy. They cannot cross the blood–brain barrier and therefore have no effect on conversion of levodopa into dopamine in the brain. However, in a substantial part of PD patients sooner or later response fluctuations occur, the therapeutic window narrows and larger doses of levodopa are necessary with a greater likelihood of side effects. These phenomena are correlated to disease duration and severity, suggesting that increased loss of dopaminergic innervation of the striatum is largely responsible^2–6^. However, recent work offered additional explanations, namely peripheral mechanisms altering the bioavailability and, accordingly, the efficacy of levodopa. For example, gut microbiota may modify levodopa’s bioavailability by expressing the enzyme tyrosine decarboxylase that can convert levodopa into dopamine, thereby reducing the amount of levodopa available for the brain^7^. We here examine another peripheral obstacle for levodopa to reach the brain, namely induced AADC activity in blood. Specifically, we evaluated the effect of chronic levodopa/PDI use on serum AADC enzyme activity.

## Results

### Group differences

**Fig. 1:**
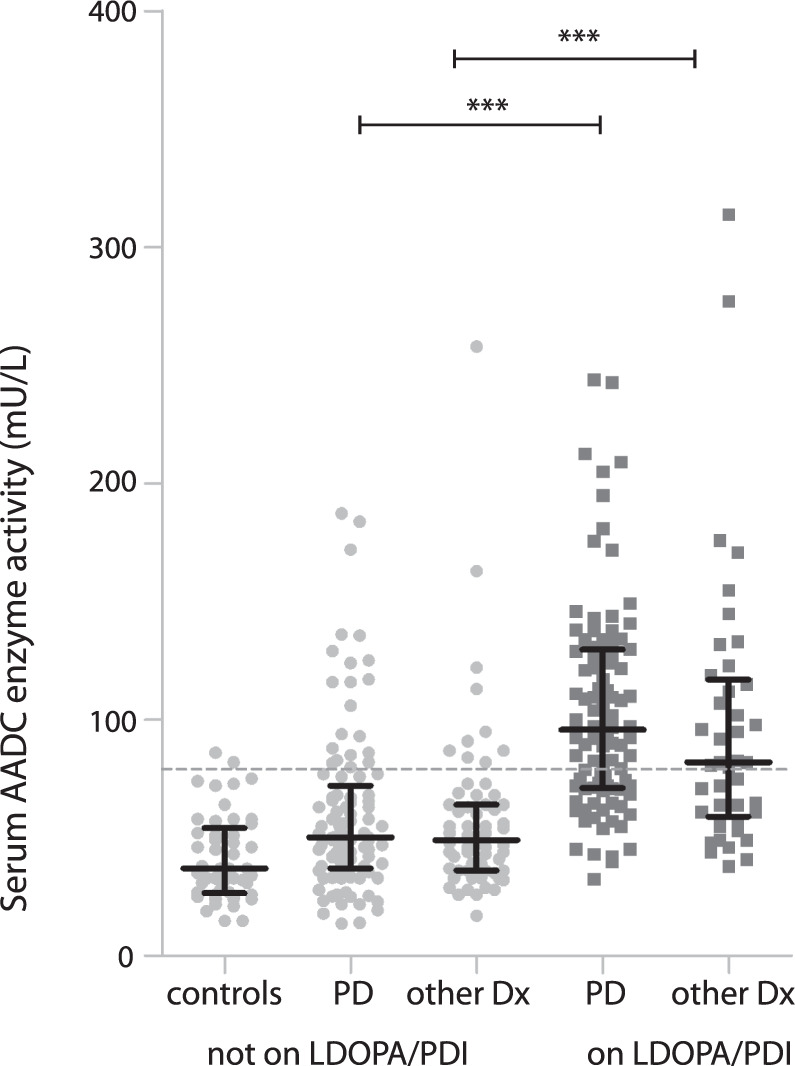
Serum AADC enzyme activity in non-neurological controls, patients with and without chronic use of levodopa/PDI. AADC enzyme activity is elevated in patients on, compared to patients not on, levodopa/PDI treatment. Median AADC activity is shown with interquartile range. Dashed line: cutoff value of AADC 79 mU/L, based on the 95th percentile of 49 controls free from neurological disease. ***The difference is significant at the *p* < 0.001 level (Mann–Whitney *U*). 1 data point is outside the axis limits: PD patient on LDOPA/PDI with AADC 663 mU/L. AADC aromatic L-amino acid decarboxylase, LDOPA levodopa, other Dx patients with other neurodegenerative or movement disorder than Parkinson’s disease, PD Parkinson’s disease, PDI peripheral decarboxylase inhibitor.

**Fig. 2:**
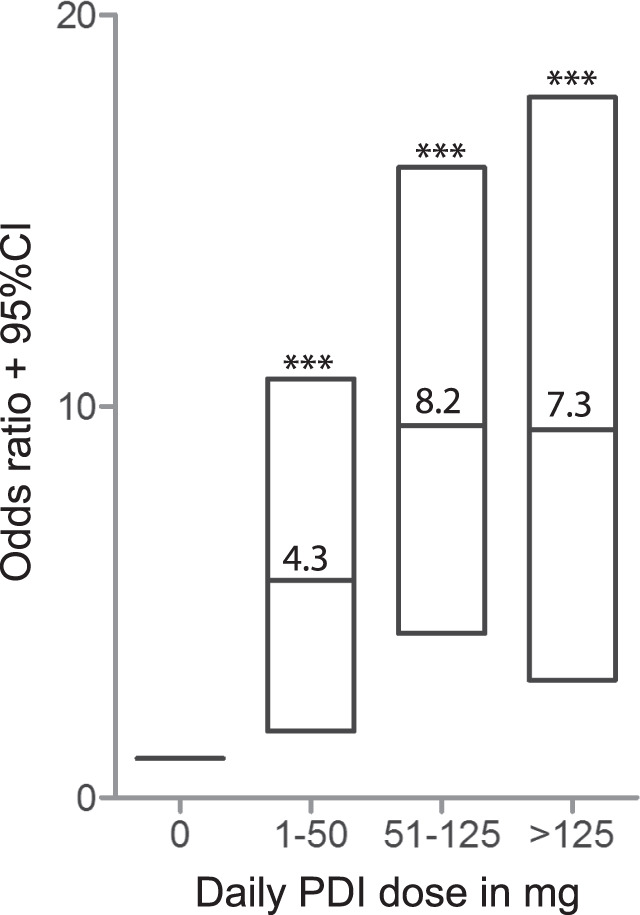
Likelihood of increased serum AADC. The likelihood of elevated serum AADC increases with daily levodopa/PDI dose. Odds ratios and 95% CI for elevated serum AADC enzyme activity (>79 mU/L) are shown per category of daily PDI dose (levodopa/PDI formulations are 4:1), analyzed with multiple logistic regression adjusted for age, gender, disease duration, and diagnosis. AADC aromatic L-amino acid decarboxylase, CI confidence interval, PDI peripheral decarboxylase inhibitor. ***significant at the *p* < 0.001 level.

Serum AADC enzyme activity was evaluated in a discovery cohort and validated in two independent cohorts of patients with PD or parkinsonism (*n* = 301) and compared between patients on levodopa/PDI (*n* = 140) vs. patients not on this medication (*n* = 161). Baseline characteristics of the study population and main results are shown in Table 1. There were 197 patients diagnosed with PD and 104 patients with another neurodegenerative or movement disorder (atypical parkinsonism (*n* = 55), secondary parkinsonism (*n* = 27), dementia (*n* = 10), tremor or dystonia (*n* = 7), and other (*n* = 5, e.g., restless legs syndrome, late onset ataxia). Median AADC enzyme activity was elevated in patients using levodopa/PDI (median 90 mU/L) compared to patients not using levodopa in all three cohorts (median 50 mU/L, *p* < 0.001). There was no significant difference between patients with PD or other movement disorders with levodopa/PDI (median 96 vs. 82 mU/L, *p* 0.29) and the same groups without levodopa/PDI (median 50 vs. 49 mU/L, *p* 0.94, Fig. 1). An elevated AADC level (>79 mU/L) was found in 62% of patients using levodopa/PDI, compared to 19% of patients without levodopa (*p* < 0.001). Patients with an elevated AADC were older, had a longer disease duration, and had a higher daily levodopa dose. Multiple logistic regression analysis showed an increased likelihood of elevated AADC for patients using levodopa (OR 6.6, 95% CI 2.8–15.6, *p* 0.001, adjusted for age, gender, diagnosis, disease duration, and daily levodopa/PDI dose). Higher daily doses of levodopa/PDI increased the likelihood of elevated AADC (Fig. 2).

**Table 1:** Baseline characteristics of Radboudumc, Kassel, and Luxembourg cohorts.

	Radboudumc^10^ discovery cohort		Kassel^11^ validation cohort		Luxembourg^12^ validation cohort		Three cohorts	
Levodopa/PDI use	Yes	No	Yes	No	Yes	No	Yes	No
Patients, *n*	27	108	60	31	50	25	137	164
Diagnosis PD/other diagnosis^a^	15/12	71/37	31/29	5/26	50/0	25/0	96/41	101/63
Age (years), mean ± SD	64 ± 9	60 ± 10	72 ± 8	70 ± 12	81 ± 6	79 ± 6	74 ± 10	65 ± 12
Gender, men/women	14/13	71/37	39/21	15/16	26/24	13/12	79/58	99/65
Diagnosis (years), median (IQR)	2 (1–4)	0 (0-1)	4 (2–7)	3 (2–6)	4 (1–7)	1 (0–3)	3 (1–6)	0 (0-2)
Hoehn and Yahr score, median (IQR)	2.5 (2–3)	2.5 (1.5–3)	4 (3–4)	1 (0-2.5)	2 (2–3)	2 (2–3)	2.5 (2–4)	2 (1.5–3)
MDS-UPDRS III score, mean ± SD	41 ± 14	35 ± 17	34 ± 16	19 ± 14	35 ± 18	41 ± 16	36 ± 17	33 ± 17
Levodopa daily dose, median (IQR)	300 (150–544)	–	450 (250–600)	–	30 (300–531)	–	388 (250–550)	–
Serum AADC (mU/L), median (IQR)	114^b^ (64–145)	52^b^ (39–75)	85^b^ (63–125)	48^b^ (37–64)	90^b^ (70–113)	42^b^ (25–59)	90^b^ (65–126)	50^b^ (36–68)
Elevated serum AADC (>79 mU/L), *n* (%)	19^b^ (70%)	22^b^ (20%)	36^b^ (60%)	4^b^ (13%)	30^b^ (60%)	5^b^ (20%)	85^b^ (62%)	31^b^ (19%)

*AADC* aromatic L-amino acid decarboxylase enzyme activity, *IQR* interquartile range, *n*/*a* not available, *PD* Parkinson’s disease, *PDI* peripheral decarboxylase inhibitor, *SD* standard deviation, *MDS-UPDRS III* Movement Disorder Society Unified Parkinson’s Disease Rating Scale part III.

^a^Other neurodegenerative or movement disorder (atypical parkinsonism (*n* = 55), secondary parkinsonism (*n* = 27), dementia (*n* = 10), tremor or dystonia (*n* = 7), and other (*n* = 5, e.g., restless legs syndrome, late onset ataxia).

^b^Differences between subgroups with vs. without levodopa/PDI use are significant (*p* < 0.001), analyzed using Mann–Whitney U test for comparison of two groups.

### Medication characteristics

There was no significant difference in AADC enzyme activity between patients with and without sustained release levodopa medication or between carbidopa vs. benserazide. Dose frequency was not different between patients with normal vs. elevated AADC. Patients with elevated AADC used more often and higher daily doses of other dopaminergic medication (Table 2). Dopamine agonists and COMT inhibitors were used more often by patients with elevated AADC (dopamine agonists 36% vs. 18%, *p* = 0.001; COMT inhibitors 12% vs. 4%, *p* = 0.004), but were unlikely to interact with AADC enzyme activity directly (AADC activity in patients with vs. without this medication was similar). Use of other comedication (MAO-B inhibitors, amantadine, serotonergic medication) was comparable.

**Table 2:** Patient characteristics in subgroups with normal and elevated (>79 mU/L) serum AADC enzyme activity.

	Normal AADC	High AADC	*p* value
Serum AADC activity range, mU/L	14–79	79–663	
Patients, *n*	185	116	
Diagnosis PD/other diagnosis^a^	114/71	83/33	NS^b^
Age (years), mean ± SD	68 ± 12	71 ± 11	0.048^c^
Gender, men/women	114/71	64/52	NS^b^
Symptoms (years), median (IQR)	2.5 (1.5–5.0)	3.3 (2.0–6.0)	0.006^c^
Diagnosis (years), median (IQR)	1.0 (0–3.0)	2.0 (1.0–5.0)	<0.001^c^
Hoehn and Yahr score, median (IQR)	2.5 (1.5-3)	2.5 (2–3)	NS^c^
MDS-UPDRS III score, mean ± SD	35 ± 18	34 ± 15	NS^c^
LEDD (mg), median (IQR)	0 (0–300)	400 (160–600)	<0.001^c^
Levodopa	0 (0–150)	300 (0–500)	<0.001^c^
Other dopa medication^d^	0 (0–0)	25 (0–185)	0.001^c^

*COMT* catechol-O-methyltransferase, *IQR* interquartile range, *LEDD* levodopa equivalent daily dose, *MAO* monoamine oxidase, *NS* not significant, *PD* Parkinson’s disease, *SD* standard deviation, *MDS-UPDRS III* Movement Disorder Society Unified Parkinson’s Disease Rating Scale part III.

^a^Other neurodegenerative or movement disorder (see Table 1).

^b^Analyzed using chi-squared test.

^c^Analyzed using Kruskal–Wallis test with post hoc Dunn’s test.

^d^Other dopaminergic medication: amantadine, COMT inhibitors, dopamine agonists, and MAO-B inhibitors.

### Dopaminergic response

Among the 96 PD patients using levodopa, a beneficial dopaminergic response (defined as (1) documented history of marked improvement of motor function after initiation of dopaminergic treatment and/or with dose increases or marked worsening with dose decreases or (2) documented history of marked on/off fluctuations, including predictable end-of-dose wearing off) was present in 71% of patients with normal AADC activity vs. 73% with elevated AADC activity (*p* = 0.17). Response fluctuations were present in 18% of the PD patients with normal AADC activity vs. 23% with elevated AADC (dyskinesias 6% vs. 9%, *p* = 0.71, and wearing off 13% vs. 19%, *p* = 0.55). These group differences were not significant. There were no differences in side effects (nausea, only documented in 17%, and orthostatic hypotension, documented in 36%) and constipation (as possible interfering factor for medication effect, documented in 35%).

## Discussion

We found that serum AADC enzyme activity is markedly elevated in patients using levodopa with a PDI compared to patients not using (levodopa with) a PDI. This AADC induction is paradoxical, since PDIs are administered in order to inhibit AADC activity, thereby increasing levodopa’s therapeutic efficacy while reducing any systemic side effects. AADC induction, as a consequence of and response to chronic levodopa/PDI use, may therefore increase the peaks and troughs in plasma levodopa levels contributing to the development of response fluctuations. This is important since pulsatile stimulation of dopamine receptors is believed to play an important role in the development of dyskinesias^2^. In the past decades, there has been a longstanding discussion as to why and how levodopa loses effectiveness. It is remarkable, however, that AADC, the enzyme that converts levodopa into dopamine, has only received minimal consideration in this context.

Serum AADC induction by PDIs was noted only once before in a small longitudinal study in 1989, but was not followed up ever since^8^. This earlier study showed that the inhibitory effect of 25 mg carbidopa ended ~90 min after oral intake. Then, AADC activity returned to baseline levels. This baseline serum AADC activity rose gradually after 3–4 weeks of levodopa/PDI use^8,9^. AADC induction could not be explained by levodopa-related enzyme induction, since intake of levodopa without PDI did not induce AADC. Moreover, it was not explained by general liver enzyme induction, since it was not observed in patients with chronic use of known liver enzyme inducers such as the antiepileptic drug phenytoin^8^. Instead, a specific induction of the serum AADC enzyme concentration as a result of compensatory autoregulation may be considered as underlying cause for our observations. The precise pathophysiological mechanism for serum AADC induction remains to be established.

One possible underlying or contributing factor might be chronic (partial) vitamin B6 depletion. Chronic treatment with levodopa and PDIs is associated with (1) partial vitamin B6 depletion, since PDIs bind irreversibly to PLP, the active form of vitamin B6, and (2) with a dysfunctional methionine metabolism, which is characterized by an increased methylmalonic acid, increased homocysteine, and deficiencies of vitamin B6, folate (vitamin B11), and vitamin B12^10,11^. Exogenous pyridoxine (vitamin B6) enhances peripheral levodopa degradation. Based on plasma levodopa levels and its metabolites, this enhancing effect of exogenous pyridoxine on peripheral levodopa degradation seems extra strong in patients with chronic levodopa use. This could be explained by chronic (partial) vitamin B6 depletion, AADC induction, or both^12^.

The paradoxical induction of AADC activity could hypothetically necessitate a gradual increase in required levodopa dosage, due to increased conversion into dopamine in blood, and negatively affect levodopa’s efficacy, inducing response fluctuations and causing side effects. PD patients in advanced disease stages may need up to tenfold higher daily levodopa doses than PD patients in early disease stages. It is widely assumed that this is due to more comprehensive dopaminergic neuronal cell death, creating a larger dopamine deficit. However, since at disease onset already 50–80% of dopaminergic neurons in the substantia nigra have degenerated^13^, it is difficult to understand that a relatively small further increase in dopaminergic cell death is solely responsible for this dramatic loss of drug efficacy. In this study, patients with high AADC activity used higher doses of levodopa and more frequently used dopamine agonists and COMT inhibitors, perhaps to compensate for the relative loss of levodopa efficacy. In line with our findings, a recent study has demonstrated that gut microbiota may also convert levodopa into dopamine even before resorption^7^. Thus, peripheral conversion of levodopa into dopamine, both in the gut and in blood, might compromise bioavailability and diminish the therapeutic efficacy of levodopa over time. There are preliminary indications that higher doses of PDI may increase the treatment effect of levodopa^14,15^.

Our study was neither designed to study all possible underlying pharmacokinetic mechanisms of AADC induction nor the clinical consequences and therefore has its limitations. First, this is a retrospective study of three cohorts, none of which was designed specifically for this purpose. Therefore, duration of levodopa use and time of blood collection in relation to last levodopa intake were neither routinely documented nor standardized. Second, AADC activity was measured only once for each patient (cross-sectionally); a longitudinal study could provide clearer perspectives onto the dynamics of AADC activity. Third, this study lacked power to detect significant differences in treatment complications in the subgroup of PD patients with levodopa/PDI, therefore implications of our findings on the management of PD patients are yet unclear.

Ideally, AADC activity should be evaluated in patients before start with levodopa and at several time points during treatment, with evaluation of diurnal fluctuations and cumulative intake of levodopa medication. Further pharmacokinetic studies addressing the complex of neurochemical responses and treatment responses after chronic levodopa use are needed to provide more insight in the clinical and therapeutic consequences of AADC induction. Future studies in this area should also measure levels of PLP and other vitamin B6 compounds, alongside with AADC activity. Moreover, studies on the clinical and biochemical effect of higher doses of PDI may be warranted.

This study provides an impetus for further research and discussion regarding the biochemical adaptations occurring after chronic administration of levodopa with PDIs. This knowledge can be exploited to optimize levodopa treatment and the development of possible novel adjuvants to benefit patients with PD.

## Methods

### Patients

Serum samples were obtained from 301 patients in total, consisting of 140 patients using levodopa/PDI and 161 patients not using levodopa/PDI. AADC enzyme activity measurements were first done in a discovery cohort, a cross-sectional analysis as part of a prospective biomarker study in patients with parkinsonism and an initially uncertain diagnosis (*n* = 135) in the Radboudumc in the Netherlands^16^. The results were validated in two large independent cohorts with prospectively collected data: (1) Kassel cohorts in Germany (*n* = 91, a biomarker study in patients referred with a working diagnosis of parkinsonism)^17^ and (2) the Luxembourg Parkinson’s Study (*n* = 75, a diagnostic and progression marker study including patients in all disease stages of PD)^18^. We aimed for a 2:1 ratio of patients with and without levodopa and PDI, matched for age and gender. In order to minimize potentially confounding factors such as diagnosis specific factors, disease duration, and duration of levodopa therapy for the first validation cohort, a heterogeneous population was selected including both PD patients and patients with other neurodegenerative or movement disorders (since PD patients on levodopa therapy are likely to have more advanced disease compared to PD patients not on levodopa therapy, which is not necessarily the case in other neurodegenerative or movement disorders). For the second validation cohort, a population of only PD patients was selected. All patients on levodopa/PDI treatment used formulations of 4:1 (levodopa vs. PDI dose). Full clinical details of these cohorts have been described previously^16–18^.

### Ethics statement

For all three cohorts, the study protocol was approved by the local medical ethics committees: “Commissie Mensgebonden Onderzoek regio Arnhem-Nijmegen” in the Netherlands; the Ethics Committee of the University of Goettingen in Germany; and the National Research Ethics Committee in Luxembourg. All participants provided written informed consent prior to enrollment.

### AADC enzyme activity measurements

Venous blood samples were obtained at the time of enrollment in the cohort study. Blood was processed at the study site via standardized procedures into serum, including rapid transport to the adjacent lab, centrifugation of the tubes, aliquoting in small standardized volumes, and storage in biobanks at −80 °C. Serum samples from Kassel and Luxemburg were sent on dry ice to the Radboud University Medical Center. Enzymatic conversion of levodopa into dopamine was determined as described previously^19^. First, a mixture of 150 μL serum and 150 μL PLP (the active form of vitamin B6 in order to activate the AADC enzyme, 0.7 mM, Merck Darmstadt, Germany) was preincubated with 900 μL phosphate buffer (167 mM, pH 7.0) containing 39 mM dithioerythritol (Aldrich, Steinheim, Germany) and 0.167 mM sodium ethylenediaminetetraacetic acid (Baker, Deventer, the Netherlands) for 2 h at 37 °C. Then, 300 μL of levodopa (20 mM) was added to be used as a substrate (for the AADC enzyme to convert into dopamine) and incubated at 37 °C. After 2 h, the enzyme reaction was ended by the addition of 120 μL perchloric acid (70%, Merck). Fifty microliters of DHBA (3,4-dihydroxybenzylaminehydrobromine, Sigma) was added as internal standard for the Sephadex isolation of dopamine. The tubes were centrifuged at 3500 rpm for 5 min. Before loading, the Sephadex G10 column was regenerated with 3 mL ammonia (26.7 mM, Merck) and 3 mL formic acid (0.03%). Subsequently, 1 mL of supernatant was loaded on a (homemade) Sephadex G10 (Sigma) minicolumn (7 cm × 5 mm). The column was washed with 2 mL formic acid (0.03%, Merck), eluted with a similar amount of formic acid, and analyzed by high-performance liquid chromatography. AADC enzyme activity was calculated (in duplicate) as the amount of dopamine formed during the reaction per minute per volume and expressed as mU/L (1U = 1 mole of dopamine formed per minute).

### Control population

AADC enzyme activity measurement was previously performed in 49 control patients (23 men, 26 women) free from neurological disease and without use of levodopa or PDI. Ages ranged from 15 to 82 years, with a mean age of 44 years and mean AADC enzyme activity of 42 mU/L (range 15–86 mU/L). In this control population, there were neither a significant difference between sexes (mean AADC activity in men 41 and women 42 mU/L, *p* 0.93) nor in age categories (mean AADC activity aged < 50 years, 42 mU/L, and >50 years, 40 mU/L, *p* 0.70). AADC enzyme activity was not correlated to age. The 95th percentile AADC enzyme activity in the control population (79 mU/L) was used as cutoff value for the patient cohorts^19^.

### Statistics

To compare independent groups, the Student’s *t* test, Mann–Whitney *U* with Bonferroni post hoc test, or Kruskal–Wallis test with Dunn’s post hoc test for continuous variables and chi-square or Fisher exact test for dichotomous variables were used. AADC enzyme activity levels of patients with and without levodopa plus PDI were compared. Differences in disease duration, disease severity (UPDRS III, H&Y), daily levodopa dose, medication use, response fluctuations, and levodopa-related side effects were evaluated between subgroups with low and high AADC. Correlations were investigated by Pearson’s or Spearman’s test as appropriate. We performed multiple logistic regression analysis to estimate the likelihood of elevated serum AADC in patients with levodopa (adjusted for age, gender, diagnosis (PD vs. other neurodegenerative or movement disorder), disease duration, and daily levodopa dose) and the likelihood of elevated serum AADC based on the daily levodopa dose (adjusted for age, gender, diagnosis, and disease duration). Data analysis was done by using SPSS Statistics 22 (IBM Corp., Armonk, NY) and GraphPad Prism 5 (La Jolla, CA).

### Reporting summary

Further information on research design is available in the Nature Research Reporting Summary linked to this article.

## Supplementary information

REPORTING SUMMARY

## Data Availability

Anonymized data will be shared on request from any qualified investigator for purposes of replicating procedures and results.
