# Supplementary material for: Peripheral decarboxylase inhibitors paradoxically induce aromatic L-amino acid decarboxylase
Source: NPJ Parkinsons Dis. 2021 Mar 19;7:29. doi: 10.1038/s41531-021-00172-z (PMC7979935; doi:10.1038/s41531-021-00172-z)
Supplement: Supplementary file 1 — REPORTING SUMMARY [file 41531_2021_172_MOESM1_ESM.pdf]

nature research

Corresponding author(s): NPJPARKD-00551

Last updated by author(s): Jan 4, 2021

Reporting Summary

Nature Research wishes to improve the reproducibility of the work that we publish. This form provides structure for consistency and transparency in reporting. For further information on Nature Research policies, see our Editorial Policies and the Editorial Policy Checklist.

Statistics

For all statistical analyses, confirm that the following items are present in the figure legend, table legend, main text, or Methods section.

n/a	Confirmed	
☐	☒	The exact sample size (n) for each experimental group/condition, given as a discrete number and unit of measurement
☐	☒	A statement on whether measurements were taken from distinct samples or whether the same sample was measured repeatedly
☐	☒	The statistical test(s) used AND whether they are one- or two-sided<br>*Only common tests should be described solely by name; describe more complex techniques in the Methods section.*
☐	☒	A description of all covariates tested
☐	☒	A description of any assumptions or corrections, such as tests of normality and adjustment for multiple comparisons
☐	☒	A full description of the statistical parameters including central tendency (e.g. means) or other basic estimates (e.g. regression coefficient) AND variation (e.g. standard deviation) or associated estimates of uncertainty (e.g. confidence intervals)
☐	☒	For null hypothesis testing, the test statistic (e.g. F, t, r) with confidence intervals, effect sizes, degrees of freedom and P value noted<br>*Give P values as exact values whenever suitable.*
☒	☐	For Bayesian analysis, information on the choice of priors and Markov chain Monte Carlo settings
☒	☐	For hierarchical and complex designs, identification of the appropriate level for tests and full reporting of outcomes
☐	☒	Estimates of effect sizes (e.g. Cohen's d, Pearson's r), indicating how they were calculated

Our web collection on statistics for biologists contains articles on many of the points above.

Software and code

Policy information about availability of computer code

Data collection	no software was used
Data analysis	Data analysis was done by using SPSS Statistics 22 (IBM Corp., Armonk, NY) and GraphPad Prism 5 (La Jolla, CA).

For manuscripts utilizing custom algorithms or software that are central to the research but not yet described in published literature, software must be made available to editors and reviewers. We strongly encourage code deposition in a community repository (e.g. GitHub). See the Nature Research guidelines for submitting code & software for further information.

Data

Policy information about availability of data

All manuscripts must include a data availability statement. This statement should provide the following information, where applicable:

- Accession codes, unique identifiers, or web links for publicly available datasets
- A list of figures that have associated raw data
- A description of any restrictions on data availability

Anonymized data will be shared on request from any qualified investigator for purposes of replicating procedures and results.

Field-specific reporting

Please select the one below that is the best fit for your research. If you are not sure, read the appropriate sections before making your selection.

☒ Life sciences ☐ Behavioural & social sciences ☐ Ecological, evolutionary & environmental sciences

For a reference copy of the document with all sections, see nature.com/documents/nr-reporting-summary-flat.pdf

Life sciences study design

All studies must disclose on these points even when the disclosure is negative.

Sample size	The laboratory measurement of interest (AADC enzyme activity measurement) was first done in a discovery cohort (n=135, a cross-sectional analysis). The results were validated in two independent cohorts with prospectively collected data. Sample size of the validation cohorts was based on the initial results in the discovery cohort (mean AADC enzyme activity and standard deviation, with 90% power and alpha of 0,05), resulting in a calculated sample size of 133. For the validation cohorts we used (1) the Kassel cohorts in Germany (n=91) and (2) the Luxembourg Parkinson's Study (n=75), resulting in a total n of 166 for the two validation cohorts, which was slightly larger than the required sample size of 133.
Data exclusions	no data were excluded from the analysis
Replication	As described above, the results of the discovery cohort were validated in two large independent cohorts with prospectively collected data: (1) Kassel cohorts in Germany (n=91) and (2) the Luxembourg Parkinson's Study (n=75).
Randomization	participants were not allocated to experimental groups because no intervention was studied, therefore randomization did not take place.
Blinding	clinical data were disclosed after completing the laboratory measurements

Reporting for specific materials, systems and methods

We require information from authors about some types of materials, experimental systems and methods used in many studies. Here, indicate whether each material, system or method listed is relevant to your study. If you are not sure if a list item applies to your research, read the appropriate section before selecting a response.

Materials & experimental systems

n/a	Involved in the study	
☒	☐	Antibodies
☒	☐	Eukaryotic cell lines
☒	☐	Palaeontology and archaeology
☒	☐	Animals and other organisms
☐	☒	Human research participants
☐	☒	Clinical data
☒	☐	Dual use research of concern

Methods

n/a	Involved in the study	
☒	☐	ChIP-seq
☒	☐	Flow cytometry
☒	☐	MRI-based neuroimaging

Human research participants

Policy information about studies involving human research participants

Population characteristics	The study population consisted of 301 patients with parkinsonism (mean age 69 +/- 12 years, 59% men), consisting of 140 patients using levodopa with a peripheral decarboxylase inhibitor (PDI) and 161 patients not using this medication.
Recruitment	Patients were recruited at three independent movement disorder clinics (Nijmegen, the Netherlands; Kassel, Germany; Luxembourg). Although these were all tertiary referral centers (with probably more severe/atypical cases), for this research question there is no risk of selection bias.
Ethics oversight	For all three cohorts, the study protocol was approved by the local medical ethics committees: "Commissie Mensgebonden Onderzoek regio Arnhem-Nijmegen'' in the Netherlands; the Ethics Committee of the University of Goettingen in Germany; and the National Research Ethics Committee in Luxembourg. All participants provided written informed consent prior to enrollment.

Note that full information on the approval of the study protocol must also be provided in the manuscript.

Clinical data

Policy information about clinical studies

All manuscripts should comply with the ICMJE guidelines for publication of clinical research and a completed CONSORT checklist must be included with all submissions.

Clinical trial registration	this study is an observational study and no clinical trial. It was therefore not registered as clinical trial.
Study protocol	study protocol for cohort 1 (Radboudumc): https://doi.org/10.1007/s00415-014-7568-4<br>study protocol for cohort 2 (Kassel Germany): https://www.sciencedirect.com/science/article/pii/S0014488608002562?via%3Dihub<br>study protocol for cohort 3 (Luxembourg): https://www.frontiersin.org/articles/10.3389/fnagi.2018.00326/full
Data collection	Cohort 1 (Radboudumc): January 2003 - December 2006, Radboudumc Center of Expertise for Parkinson & Movement Disorders, Nijmegen, The Netherlands.<br>Cohort 2 (Kassel Germany): January 2003 - December 2006, Departments of Psychiatry and Neurology at the University of Goettingen, Germany, at the Paracelsus-Elena-Klinik, Kassel, Germany<br>Cohort 3 (Luxembourg): January 2018 - December 2018, Clinical and Experimental Neuroscience, Luxembourg Centre for Systems Biomedicine (LCSB), University of Luxembourg, Esch-sur-Alzette, Luxembourg.
Outcomes	Serum AADC enzyme activity (amount of dopamine formed during the enzyme reaction per minute per volume and expressed as mU/L) was evaluated in patients with Parkinson’s disease or parkinsonism and compared between patients on levodopa with peripheral decarboxylase inhibitor versus patients not on this medication
